# Supplementary material for: Are carbon emissions trading and green financial instruments synergistic? -Comprehensive quantitative research based on content analysis
Source: PLoS One. 2024 Mar 7;19(3):e0298601. doi: 10.1371/journal.pone.0298601 (PMC10919601; doi:10.1371/journal.pone.0298601)
Supplement: S1 Data — (ZIP) [file pone.0298601.s001.zip › Data/地级市Z_F工作报告.zip!/╣≤╓▌/░▓╦│╩╨/└┤╫╘╣1⁄2╓┌║┼í╛┬φ┐╦╩2╛▌═°í┐_2003.docx]

各位代表：

现在，我代表市人民政府向大会作工作报告，请予审议，并请各位政协委员提出意见。

2002年政府工作回顾

2002年，在省委、省政府和市委的正确领导下，在市人大、市政协的监督和帮助下，市人民政府坚持以邓小平理论和“三个代表”重要思想为指导，全面贯彻执行党中央、国务院的各项方针政策，认真履行全市各族人民赋予的职责，以创造新业绩，迎接党的十六大胜利召开为主线，经过全市上下的共同努力，较好地完成了全年经济社会发展各项目标任务。

国民经济保持较快增长，综合经济实力得到增强。全年国内生产总值70.73亿元，同比增长9.13％。其中第一产业增加值17.54亿元，同比下降7.17%；第二产业增加值29.74亿元，同比增长22.48%；第三产业增加值23.45亿元，同比增长8.80%。三次产业构成为24.8:42.0:33.2。财政总收入8.77亿元，同比增长16.95％，其中地方财政收入5.08亿元，同比增长19.85％，分别上了新台阶。全市经济增长速度分别高于全国、全省平均水平，继续在九个市州地排位靠前。

固定资产投资稳步增长，基础设施建设进展顺利。抢抓国家实施积极财政政策和西部大开发、新阶段扶贫开发的战略机遇，加大投资力度，扩大有效需求，带动经济增长。全社会固定资产投资规模达21.11亿元（不含跨区项目），同比增长13.13％，固定资产投资拉动经济增长5.5个百分点。

以清（镇）镇（宁）高速公路和关（岭）兴（义）、水（城）黄（果树）高等级公路以及安顺黄果树机场等为重点的交通基础设施建设，以安顺电厂二期、引子渡电站等为重点的能源建设，以王二河水库、安西灌区等为重点的水利建设，以城市道路、供水管网改造为重点的城镇基础设施建设和以退耕还林还草为重点的生态建设等一批重点项目顺利建成或正在实施。“十件实事”基本完成。

完成了《安顺市城市总体规划》、《安顺市市域城镇总体规划》、《安顺市中华东、西路街区控制性详细规划》、《安顺市中华东、西路街区修建性详细规划》、《安顺市若飞广场规划》、《体育中心规划》、《黎阳高新区综合服务组团控制性规划》、《龙泉路地段控制性详细规划》的编制工作；各县区《城镇总体规划》正按要求进行第三轮修编。《安顺市历史街区保护规划》已通过评审。完成了贵州省旅游总体规划安顺章节相关资料的收集整理编写工作；完成了龙宫风景名胜区中心景区、漩塘景区规划编制工作，并通过评审；完成了紫云格凸河穿洞风景名胜区、关岭花江大峡谷风景名胜区总体规划和镇宁普里山风景名胜区可行性研究报告编制工作，并通过论证。完成了全市6个县区、54个重点乡镇、991个扶贫工作重点村的扶贫规划。通过加强规划工作，促进了基础设施建设，带动了整体经济的发展。

结构调整进一步优化，经济增长的质量和效益明显提高。农业产业结构调整取得进展。农业科技水平不断提高，优质农产品比例上升，推行“一乡一特、一村一品”，名特优经济作物面积扩大，实现了产量、质量、效益同步增长。畜牧业发展步伐加快，以牛改为重点的畜禽品种改良工作进展顺利，畜牧业产值占农业总产值的37.1％。以贵黄公路沿线产业带为代表的一批种养业基地建设初具规模。以退耕还林还草为重点的生态建设步伐加快。关岭自治县组织民兵预备役官兵参加退耕还林的经验得到推广。对“牛来香”实业有限公司、安顺酱菜厂等一批龙头企业在市场开发、技术改造、资源培植、资金扶持等方面给予帮助，企业进一步发展。乡镇企业继续保持良好发展势头。产业结构调整促进了农村经济发展，减少了自然灾害带来的损失。

工业结构逐步优化。以构建能源、化工、制药、冶金、食品、机械制造等工业支柱为目标，延长产业链，带动了煤炭、运输、种植等其它行业的发展。突出特色，树立品牌意识，发挥“红蝶”钡盐、南风奇强、金星王啤酒等品牌效应，产业规模有新扩展。实现工业增加值11.16亿元，同比增长15.1％。非公有制经济发展加快。全市规模以上非公有制企业完成工业总产值14.84亿元，同比增长23%，总产值占市县属规模以上工业经济的71.75％。

以旅游业为龙头的第三产业发展态势良好。全年接待国内外游客139.9万人次，同比增长11％；营业收入3.33亿元，同比增长11.1％。金融形势运行平稳，全市金融机构各项存款余额84.08亿元，同比增长22.99%；各项贷款余额91.28亿元，同比增长33.04%。房地产业发展势头强劲，商贸、保险、交通运输、邮电通信等服务业稳步发展，信息、会计、法律服务等行业发展加快，餐饮业呈快速发展势头。第三产业整体水平有新提高。

改革开放取得新进展，人民生活进一步改善。扩大农村税费改革试点工作成效明显，政策内农民负担减幅为52.86%，大大超过预定税改减负目标；结合税费改革工作，切实提高了村干部待遇。国有企业改革取得成效。结合城市建设，促进企业“退二进三”，盘活了存量资产。财政体制改革逐步深化，财政监督力度加大，提高了政府办事能力。社会保障体系改革试点工作进展顺利。“两个确保”100%的成果得到巩固，做到了国有企业离退休人员基本养老金100％按时足额发放，全面实现养老金社会化发放目标；医疗保险体制改革顺利推进，登记参保人数已达8万余人，实施缴费运行人数达5万多人。采取多种形式、多种渠道大力促进就业和再就业，城镇登记失业率控制在5%。全年劳务输出35633人次，劳务输出人员汇回资金达1.95亿元。整顿和规范市场经济秩序，特别是整治集贸市场、加油站、旅游市场和建筑市场成效显著。成立市商务中心，推行“一站式”办公、“一条龙”服务；组织开展了“走访百家企业活动”，掌握了投资环境、机关服务、企业经营情况及存在的问题，并责成相关部门进行整改，使投资环境和企业经营外部环境逐步得到改善。认真贯彻落实《党政领导干部选拔任用工作条例》，实行市级机关中层干部跨部门公开竞争上岗，干部队伍结构得到优化。对市直部门、单位科级以上干部进行了WTO知识培训。同青岛市对口帮扶工作进一步加强，与北京市房山区结成友好城市，对美国兰开斯特县进行了友好访问。

人民生活进一步改善。城镇居民人均可支配收入5206元，同比增长7.6％；农民人均纯收入1414元，同比增长2.3％；社会消费品零售总额25.56亿元，同比增长10.4％；又有4.08万农村贫困人口越过温饱线。

实施科教兴安和可持续发展战略，各项社会事业全面发展。科技工作在创新中得到发展。共争取国家和省科技项目9项；镇宁自治县被列入“十五”期间第一批国家级“星火西进示范县”；科技体制改革和科研成果推广应用取得良好效果，制定了《安顺市科学技术进步奖励实施细则》，科技成果管理进一步规范化、法制化。

教育事业稳步发展。“两基”攻坚工作进展顺利，全市“普九”人口覆盖率89.11%，非文盲率97.3%；普定县通过省“两基”评估验收。“中小学危房改造工程”和第二期“义教工程”进展顺利。贵州师范大学安顺学院正式挂牌，组建了安顺职业技术学院，完成教育部对安顺电大“人才培养模式改革和开放教育试点项目”的中期评估工作。

加强农村基层计生网络建设，基本实现“村为主”，人口出生率和自然增长率分别控制在18.7‰和11.7‰以内，同比分别下降0.76和1.21个千分点。生态建设和环境保护得到加强。文化、广播电视、卫生、体育、民族、宗教、史志、档案等工作都取得了较好成绩。

精神文明和民主法制建设得到加强，维护社会稳定工作取得成效。继续实施安顺形象工程，巩固取缔城区违规占道经营成果，加强对城市市场、道路、交通等公共服务设施的管理，深入开展“卫生与秩序”主题创建活动和“文明大道”创建活动，对贵黄公路等通往旅游景区的公路沿线公（坟）墓进行了集中整治。全面推行厂务、村务和县以上行政机关政务公开工作。认真执行人大及其常委会决议，做好政府组成部门主要负责人向人大常委会的述职工作，加强与政协的联系。全年共办复市人大代表建议、批评和意见63件，市政协委员提案102件。

加强社会治安综合治理，认真落实维护稳定的领导责任制，正确处理人民内部矛盾，防止酿成或妥善处置了群体性事件，尤其是处理好重点工程移民、征地拆迁以及城市建设中存在的突出问题，把各种矛盾和问题化解在萌芽状态。深入开展严打整治斗争，初步建立“打防控”一体化治安防范格局，切实维护了社会政治稳定。

2002年我市经济社会发展取得的成绩，是全市上下认真实践“三个代表”重要思想，抢抓机遇，艰苦奋斗，扎实工作的结果。在此，我代表市人民政府，向全市各族工人、农民、知识分子和干部，向各级人大、政协和各民主党派、工商联、人民团体和无党派爱国人士，向人民解放军、武警官兵、政法公安干警和驻安单位，向各位老领导、老同志，向所有关心、支持、参与我市建设与发展的社会各界人士表示衷心的感谢，并致以崇高的敬意！

在充分肯定成绩的同时，必须清醒地看到，经济总量小、经济结构不合理、基础设施建设相对落后、劳动者素质偏低等，仍然是制约我市经济社会发展的突出矛盾。在前进的道路上还存在诸多困难和问题：农业基础脆弱，抵御自然灾害的能力不强；农业产业化经营水平低，农民增收渠道狭窄的状况没有得到明显改善；结构调整、培育新经济增长点的工作力度还需进一步加大；一些企业生产经营的内在活力、市场竞争力和创新能力不强，经济效益与产值增长不同步；解决城市就业和再就业工作难度大；部分地区受灾群众和城市低收入家庭生活困难；投资环境、市场经济秩序、社会治安综合治理、安全生产等方面还存在不少亟待解决的问题。对此，我们必须认真对待，继续采取有效措施切实加以解决。

2003年政府工作主要任务

党的十六大描绘了全面建设小康社会的宏伟蓝图，市委一届五次全体（扩大）会议提出了我市建设小康社会的奋斗目标。2003年是深入贯彻落实党的十六大精神的第一年，是我市全面建设小康社会的开局之年，做好今年的政府工作意义重大。各级政府要继续深入贯彻落实党的十六大、省第九次党代会和市委一届五次全体（扩大）会议精神，切实把思想统一到十六大精神上来，把力量聚集到全面建设小康社会的奋斗目标上来，进一步激发全市各族人民建设小康社会的激情，抢抓机遇，加快发展，增强使命感、责任感和紧迫感，以更加务实的精神，更加扎实的工作，力争2003年的工作取得更大成效。

2003年政府工作的总体要求是：以邓小平理论和“三个代表”重要思想为指导，全面贯彻党的十六大和省第九次党代会精神，按照市委一届五次全体（扩大）会议部署，把加快发展作为政府工作第一要务，紧紧抓住西部大开发、新阶段扶贫开发的机遇，深化改革，扩大开放，全面推进经济结构调整，努力扩大固定资产投资规模，力争以交通为重点的基础设施建设再上一个新台阶，积极发展农业和农村经济，搞好扶贫攻坚，加快工业发展，大力发展以旅游业为龙头的第三产业，做好就业和再就业工作，提高城乡居民生活水平。加强社会主义精神文明和民主法制建设，推进各项社会事业全面进步。团结奋进，保持稳定，千方百计确保经济持续快速增长的好势头，以优异成绩创造全面建设小康社会的良好开局。

2003年国民经济和社会发展的主要预期目标是：（1）国内生产总值增长10%以上，其中第一产业增长7%左右，第二产业增长12%，第三产业增长11%。（2）全社会固定资产投资增长12%以上。（3）社会消费品零售总额增长10%。（4）财政总收入和地方财政收入分别（同口径）增长10%以上。（5）农民人均纯收入实际增长5%，城镇居民人均可支配收入实际增长6%。（6）城镇登记失业率控制在4.5%左右。（7）人口出生率和人口自然增长率控制在18‰、11‰以内。

全面完成今年的各项目标任务，需要我们坚持以发展为主题，以结构调整为主线，以改革开放和科技进步为动力，以提高人民生活水平为根本出发点，努力做到发展要有新思路，改革要有新突破，开放要有新局面，各项工作要有新举措。为此，我们将着力抓好以下工作：

（一）进一步扩大投资和启动消费，努力保持经济持续快速增长的良好势头

投资对我市经济增长具有决定性作用，要以大项目带动大发展。围绕国家和省投入的重点，把项目前期工作作为争取投资的关键，切实抓紧抓好。积极配合有关部门做好黄（桶）织（金）铁路、黔中水利枢纽、安顺电厂三期、光照电站、黄家湾水库、国际射电天文望远镜落户普定尚家冲等重点工程的前期工作。突出在县际公路和经济路以及水利设施、生态环境建设、改善农村生产生活条件、能源建设、产业结构调整、旅游资源开发、企业技术改造、科技教育、扩大城镇就业等方面抓紧准备一批大项目、好项目。增加对项目前期经费的投入，积极探索前期工作市场化的路子，做到开工一批、论证一批、储备一批。今年市财政预算内的贴息和补助资金在去年规模基础上适度增长，按照集中财力办大事的原则，根据实际需要和使用情况对资金投入进行调整，保证贴息资金用得出、用得好。

加快推进投融资体制改革。在启动社会投资、拓宽资金来源和融资渠道的同时，大胆举债，加快基础设施建设与城市改造步伐。在积极争取国家和省增加对我市投资的基础上，切实把启动民间投资和吸引外资作为一项重要的战略措施，尽快形成以市场推动为主的固定资产投资增长机制。加快市政基础设施投资管理体制改革，鼓励采用特许经营权转让、收费权转让、土地出让等多种方式，吸引各方面资金投资公益性基础设施建设。通过参股、控股、煤电联营等方式，积极吸引市外资金与我市合资办电、合资办矿、合资办药等，大力推进投资主体多元化。努力保持房地产等民间投资快速增长的良好势头。

围绕实施西部大开发战略，抓好重点项目建设。协调做好清（镇）镇（宁）、镇（宁）胜（境关）高速公路和关（岭）兴（义）、水（城）黄（果树）高等级公路建设的服务工作，实施好平（坝）引（子渡）、普（定）镇（宁）、紫（云）黄（果树）、镇（宁）贞（丰）等县乡公路油路改造工程；努力争取和启动农村200个通村公路建设；开通安顺黄果树机场客货运输业务。继续做好安顺电厂二期投产装机60万千瓦、引子渡电站投产装机24万千瓦、“黔电送粤”输变电工程等省重点建设项目的协调服务工作；实施好第二期农网改造项目，加快城乡电网同价进程。完成安顺大桥、市区供水管网改造、五县城引水工程等建设项目；开工建设安顺垃圾无害化处理、塔山广场、东西大道改扩建、龙泉小区开发等工程。确保完成退耕还林和荒山造林各17万亩的任务，按照“林权是核心、给粮是关键、种苗要先行、干部是保证”的要求，切实落实林草权属、管护责任和钱粮补助，充分调动农民造林的积极性；认真总结和推广在退耕还林还草方面积累的经验，特别是关岭自治县组织民兵预备役官兵参加退耕还林的经验，切实提高退耕还林的效率和质量；完成紫云自治县珠江流域和普定县长江流域水土保持治理工程；做好“珠防”和“天保”等工程，扩大封山育林面积；做好石漠化治理的相关工作；抓好农村沼气池建设，大力推广节柴省煤灶。实施好王二河水库工程，加快安西灌区、虹山水库除险加固暨贯城河治理、油菜湖水库除险加固等工程建设进度。认真做好节水示范项目，实施好“渴望工程”、“三小”水利工程和灌区改造工程。

加强重点项目管理，切实防止低水平重复建设和无效投资。全面加强重点项目资金、工期和质量的监督管理。坚决制止盲目引进和重复建设规模小、污染大、技术水平低、浪费资源的项目；坚决依法关闭“五小”企业，不准以任何理由恢复已淘汰的落后生产能力。切实提高配套资金到位率，认真研究解决财政困难县配套资金难以落实的问题。以交通建设为重点，改变投、建、管、用四位一体的管理方式。

改善消费环境，支持银行扩大消费信贷，培育和增强城乡居民的消费能力，进一步发挥消费需求对经济发展的带动作用。落实增加广大人民群众特别是低收入群众收入的各项政策措施，切实提高社会消费能力。做好今年国家将增加机关事业单位职工工资及离退休人员离退休金各项准备工作，确保调资政策按时兑现。认真搞好经济适用住房建设，启动廉租房建设前期工作，解决城市贫困居民的住房困难。整顿和规范教育、医药、住房等方面的不合理收费。鼓励租赁消费等新的消费方式，促进居民扩大汽车、旅游、教育、信息等方面的消费。

在去年工作的基础上，市委、市人民政府今年要再办十件实事：（1）确保农民人均纯收入实际增长5％以上。（2）开通安顺黄果树机场客货运输业务。（3）完成平（坝）引（子渡）公路、普（定）镇（宁）公路油路改造。（4）完成安顺大桥建设。开工建设市区中华东路、中华西路改扩建工程。开工建设市区塔山广场。改造市区运输路。（5）解决农村 8200户、4.4万人的饮水困难。完成第三期农村沼气池建设任务。（6）完成市区垃圾无害化处理50％的工程进度。完成市区贯城河治理一期工程。（7）确保完成县城城区电网改造工程计划。（8）举办油菜花旅游节、格凸河风景区首游式、黄果树瀑布节暨蜡染艺术节。完成黄果树新城基础设施建设工程。（9）改造和建设农村广播电视卫星收转站170 个。新建和扩建中小学校27所。建成3个疾病预防控制中心。完成全市乡镇计生服务站和村计生服务室建设。（10）新建经济适用住房5万平方米。

（二）大力推进农业结构调整和产业化经营，千方百计增加农民收入

以解决好“三农”问题为重点，以增加农民收入为中心，全面推进农业和农村工作。继续巩固农业基础地位，牢固树立抗灾夺丰收思想，把稳定提高粮食生产能力作为经济工作的一项重要任务来抓，力争今年粮食总产量恢复到70万吨以上。加强农业先进适用技术推广和农村基础设施建设，切实提高农业综合生产能力。继续加大种植业结构调整力度，大力推行“一乡一特、一村一品”，扩大优质农作物种植面积。引导农民发展优质油菜、特色蔬菜、烤烟等经济作物；充分发挥安顺茶叶的品牌效应，加强茶叶基地建设，不断扩大种植规模，强化产、供、销服务。以退耕还林还草为契机，在确保生态效益的前提下，把还林与还竹、还药、还果结合起来，积极发展后续产业，确保退耕农户有稳定的收入来源。把畜牧养殖业作为农业结构调整的突破口，认真实施畜牧业发展“双十亿工程”。加强以牛改为重点的畜禽品种改良工作。稳定发展生猪和大宗禽蛋生产，突出发展肉牛、肉羊和灰鹅等草食畜禽养殖，加快发展奶牛和冷水鱼类养殖，努力提高畜牧渔业产值在农业总产值中的比重。切实搞好畜禽疫病防治工作，确保畜禽产品卫生、安全。支持普定县做好省级农业结构调整试点工作。大力发展乡镇企业。重点发展劳动密集型产业和城乡服务业；继续发展矿产品加工、建材等行业；积极引导乡镇企业参与农业产业化经营。

认真落实省委、省政府关于推进农业产业化经营的各项政策措施，切实用好扶持资金，积极引进一批有品牌、有实力、带动作用强的优势企业到我市从事农业产业化经营。重点扶持一批龙头企业做大做强。大力开拓农产品市场。搞好幺铺肉牛屠宰交易市场和断桥蔬菜农副产品批发市场的改扩建工作。深化农产品流通体制改革，建立健全农村社会化服务体系。大力发展订单农业；发展农产品加工、保鲜、贮藏、运输等行业；充分发挥农经网的信息服务功能；支持行业协会、专业合作社等农村经济合作组织和农业产业化中介组织发展，加快形成龙头企业与农户之间合理的利益联结机制，提高农民进入市场的组织化程度；继续深化供销合作社改革，充分发挥其在农业产业化经营中的作用。

依靠科技进步和结构调整，积极探索加快农民脱贫致富的新思路、新办法，大力推进新阶段扶贫开发，努力提高贫困人口收入。突出抓好贫困乡、极贫村和特困户的扶贫开发，实行领导干部包乡包村扶贫责任制，确保扶贫济困的措施真正落实到村到户。继续推进麻山地区的扶贫攻坚，切实加大花山地区新阶段扶贫开发力度。改善农村生产生活条件，抓好农田、水利、农村道路和电网、沼气、通信、广播电视等方面的基础设施建设。管好用好各类扶贫资金，严格执行项目管理，全面推行报账制度，提高资金使用效益。加强农村基层组织建设，把一批有事业心、能够带领群众脱贫致富的能人充实到村级领导班子中去。力争有4万农村贫困人口越过温饱线；完成特困人口3200人的易地搬迁。一手抓扶贫，一手抓促强，支持西秀区建设经济强区，支持有条件的县参加经济强县建设。按照分类指导的原则，实事求是地制定小康建设规划，积极推进小康建设进程。

加快城镇化进程是推进农业和农村经济发展的重要途径。继续做好小城镇建设的规划编制和修编工作。加强小城镇道路、供水、供电以及污水和垃圾处理等基础设施建设，不断完善服务功能。妥善解决好小城镇建设用地，通过市场机制对城镇土地资本进行重组，实现土地资本效益最佳化。把小城镇建设同乡镇企业和农村服务业发展结合起来，引导乡镇企业向小城镇集中连片发展。支持紫云猫营、普定白岩等工业园区发展，支持以各县区城区为主的中心区域加快发展，围绕交通大通道和重大项目建设，加快规划和发展配套产业，逐步形成各具特色，各展所长的经济区和产业带。进一步深化城镇户籍制度、社会保障制度和管理体制等方面的改革。加大农村劳动力输出力度，积极引导农村富余劳动力向非农产业和城镇转移。

（三）加快工业发展，促进结构调整迈出新步伐

走新型工业化的道路，继续壮大现有优势产业，大力发展新兴产业和高新技术产业。在发展优势产业方面，一是加快机械工业发展。抓住世界性制造业转移的机遇，帮助和支持军工企业加快“军转民”步伐，加大改革改制力度，充分发挥军工企业的人才、技术、设备、管理等优势，促进和带动地方经济发展，努力形成我市新的经济增长点。二是大力发展原材料工业。利用煤炭企业市场发展较好的机遇，积极发展煤化工。加快安顺煤炭资源的详查、精查工作和煤炭工业发展规划的编制，走内外结合、煤电联营的路子，建立完善的电煤供应体系，切实做好相关服务工作。在重点抓好平坝煤矿、普定普盛煤矿、关岭坡贡煤矿，配合做好大洞口煤矿等一批大中型煤矿建设的同时，抓好现有煤矿的技改扩能。三是抓好电化学工业发展。继续抓好普定伟泰工业硅生产，促进安顺双牌铝业二期电解铝工程开工。在新兴产业和高新技术产业方面，大力发展民族药，通过联合重组，增强研发能力和市场竞争力。努力为药业企业创造良好的环境，帮助企业加快GMP认证步伐，确保未进行GMP改造的制药企业在国家规定的时限内完成改造。坚持用先进技术改造传统产业，抓好一批重点技改项目。支持红星发展不溶性硫生产线和硫脲生产线项目、安顺酱菜厂新增1.3万吨特色食品加工、华泰山药食品加工、贵州灯泡厂大输液瓶、金星啤酒厂二期工程、“牛来香”年产1000吨肉类深加工、普定明达水泥厂日产1000吨干法回转窑水泥生产线等技改项目实施。推进国民经济和社会信息化与高新技术产业发展相结合。

充分运用经济手段推进结构调整，特别是做好电价调整的相关工作，处理好鼓励优势产业发展与限制落后生产能力的关系。

（四）以旅游业为龙头，加快发展第三产业

以创建中国优秀旅游城市为目标，加快安顺旅游经济区建设。树立“大旅游、大市场、大产业”观念，认真分析和整合资源，加强总体设计和组织，加快发展自然风光和民族文化相结合的旅游业，逐步推进由单一的观光旅游向参与性强的民族风情旅游、历史文化考察、休闲度假、购物、探险等多样化旅游发展。以全省总体规划为依据，抓紧制定各景区的详细规划。深化旅游管理体制改革，推行有条件的景区景点实行特许经营和经营权转让；抓紧完成《贵州省黄果树风景名胜区管理办法》、《贵州省黄果树风景名胜区门票监管办法》，充分发挥黄果树风景名胜区旅游龙头的带动作用。加快旅游基础设施建设步伐。加快黄果树、龙宫国家级风景名胜区深度开发，开工建设王二河水库向黄果树补水工程，加快紫云格凸河、关岭花江大峡谷、普定夜郎湖等省级风景名胜区开发步伐，帮助和支持平坝天龙、西秀区云峰八寨等一批新旅游“亮点”加快发展。以清镇高速公路和普镇、紫黄公路等通往重点景区的旅游公路建设及改造为重点，大力改善旅游设施。以民居旅馆、旅游客运为重点，切实提高旅游接待能力。加快推进旅游标准化工作，加强对旅游从业人员的培训和资格认证，着力提高旅游业服务质量。对通往重点旅游区公路沿线和旅游城镇环境进行综合整治，在解决黄果树、龙宫风景名胜区存在的突出问题上取得新突破。在成功举办油菜花旅游节的基础上，办好格凸河风景区首游式、黄果树瀑布节暨蜡染艺术节、屯堡文化节。进一步加大旅游宣传促销力度，确保全市旅游总收入增长30%以上。

努力营造有利于服务业发展的良好环境。加快建立健全促进服务业发展的协调服务机制，按照政府引导、社会投入的原则，集中力量建设和改善发展服务业必须的基础设施，加快推进服务业实现市场化、社会化、产业化。以扩大就业为主攻方向，加快社区服务、餐饮、商贸流通等就业容量大的服务业发展，在有条件的地方建立和完善社区服务中心。围绕扩大内需和服务性消费，大力发展教育、文化、房地产以及医疗卫生、文化娱乐、体育等需求潜力大的服务业；以发展现代服务业为重点，积极发展中介服务、金融保险、现代物流、信息服务等新兴服务业。

（五）以国有企业改革为中心，深化各项改革

深化国有企业和国有资产管理体制改革。按照建立现代企业制度的要求，继续推进规范的公司制改革和股份制改革，完善法人治理结构，建立有效的监督机制。深化分配制度改革，逐步确立劳动、资本、技术、管理等生产要素按贡献参与分配的机制。对困难企业进行认真排队，实行“一厂一策”，协调解决已改制企业存在的困难和问题，认真做好企业的政策性破产和改革改制与资产重组工作。认真贯彻落实《中小企业促进法》，按照“有所为、有所不为”的原则，以改革改制为核心，采取多种形式放开搞活国有中小企业。根据中央、省统一部署，稳步推进国有资产管理体制改革，建立健全国有资产管理机构和机制，确保国有资产保值增值。

深化多种经济成份共生繁荣试验区改革，鼓励各类生产要素进入非公有制领域，增强非公有制经济实力。充分发挥非公有制经济在促进经济增长、扩大就业和活跃市场等方面的重要作用。把推进国有企业改革与加快非公有制经济发展结合起来，毫不动摇地巩固和发展公有制经济，着力提高国有经济的竞争力和控制力，支持和帮助多种形式的集体经济发展；毫不动摇地鼓励、支持和引导非公有制经济发展，在市场准入、技术改造、信用担保、上市融资等方面实行非公有制企业与国有企业同等待遇，为非公有制经济发展创造公平的竞争环境。依法保护非公有制经济合法权益和私有财产，依法加强监督和管理，促进非公有制经济健康发展，使非公有制经济在国民经济中的比重有明显提高。鼓励引导外资和民营资本通过参股、控股等形式，参与国有企业的改革、改组和改造，提高国有经济整体素质。

认真抓好农村各项改革。继续巩固和完善农村税费改革工作，加强对改革后农民负担的监督检查，认真落实“三个确保”，以“稳负担、防反弹、保运转”为目标，进一步完善和深化相关配套改革，使农村税费改革工作真正达到“三个经得起检验”的标准。有条件的地方，可按照依法、自愿、有偿的原则，积极稳妥地推进土地承包经营权的合理流转。按照我省粮食流通体制改革方案，加快推进国有粮食购销企业改革步伐，健全和完善粮食市场体系，全面放开粮食购销市场，确保改革顺利推进。抓好对农民补贴方式的改革工作。

切实加大行政管理体制改革力度。用改革的办法规范政府管理行为，深化行政审批制度改革，简化审批程序，规范设置审批项目，加强审批服务，建立行政审批责任追究制；抓紧解决部分部门职能交叉的问题。进一步把政府职能转到经济调节、市场监督、社会管理和公共服务上来，增强政府对经济发展的调控能力。在巩固政府机构改革成果的基础上，积极稳妥地推进事业单位人事管理制度改革。

积极推进金融体制改革。按照国家部署，协调金融部门切实做好金融改革有关工作，充分发挥金融对经济发展的支持作用。规范发展城市信用社，增强对我市经济发展特别是中小企业、非公有制经济发展的服务功能。深化农村信用社改革，明确产权关系，强化约束机制，增强为农业和农村经济服务的功能。

（六）进一步改善投资环境，提高对内对外开放水平

把改善投资环境作为扩大开放的基础工程来抓。根据国家产业政策和投资方向，积极调整利用外资重点，扩大招商引资工作实效。积极探索和采用风险投资、重组并购等方式吸引外资，不断扩大全市利用外资规模，提高利用外资质量。认清自身优势，进行科学系统策划，充分发挥招商引资机构的作用，变“守株待兔”型招商为“目的地”招商，使招商引资工作成为经济社会发展的助推器。按照十六大提出的西部地区要“着力改善投资环境”的要求，一方面要加大城市建设和管理工作力度，完善城市功能，提升城市品位；另一方面要改善投资软环境，树立安顺开放、文明、诚信、友好的新形象。针对当前我市投资环境存在的突出问题，进一步加大整治力度，严肃查处“吃、拿、卡、要”、推诿扯皮、顶着不办、延时误事的行为，增强开放意识、市场意识和规则意识。结合改革行政管理体制和行政审批制度，完善投资服务体系，规范发展各类中介组织；全面清理和废止与国家现行法律和政策相违背的、不适应改革开放需要的“土政策”；逐步建立企业和个人信用等级评级管理办法和信用不良行为的警示惩戒制度，增强全社会的信用观念。整顿和规范市场经济秩序，继续严厉打击制售假冒伪劣商品的不法行为，下决心抓好“食品放心工程”和“药品放心工程”；大力开展集贸市场、加油站、旅游市场、建筑市场、房地产市场等专项整治；坚持标本兼治，建立和健全市场准入制度；加强法制建设，严格执法，热情服务。创造招商引资良好的法制环境、诚信环境和服务环境。

充分发挥安顺经济技术开发区的机制和区位优势，使其在环境建设和环境改善方面走在全市前头，在进一步的招商引资上实现突破，进而推进全市招商引资工作有明显进展。积极支持黎阳高新工业园区和西秀新区加快基础设施建设和“筑巢引凤”进度，推进各项工作加快发展。

深入研究加入世贸组织对我市经济发展的影响，学会运用世贸组织规则和贸易争端解决机制，加大对我市相关产业和产品的合理保护。进一步加强与青岛市、北京市房山区和美国兰开斯特县等地的合作与交流，把对内对外开放提高到新水平。

（七）认真做好就业和再就业工作，继续完善社会保障体系

坚持“劳动者自主择业、市场调节就业、政府促进就业”的方针，以净增就业岗位、落实再就业政策、加大再就业资金投入和解决困难群体就业为重点，把改善创业环境和增加就业岗位作为政府的重要职责，纳入国民经济和社会发展计划并列入目标考核。通过大力发展劳动密集型产业、开拓社区就业、促进劳务输出、采取灵活多样的就业方式等措施，努力增加就业岗位。全面落实在小额信贷担保、税费减免、社会保险补贴、就业援助、主辅分离等方面促进再就业的优惠政策。完成对全市下岗失业人员发放《再就业优惠证》的工作。切实搞好再就业服务，加大再就业培训和职业介绍工作力度；引导下岗职工转变择业观念，鼓励和提倡自谋职业、自主创业；加快各级劳动力市场建设，建立劳动保障“一站式”服务体系，建立失业预警机制和城镇劳动力抽样调查制度。高度重视和加强对高等院校毕业生就业的指导和服务。

继续巩固“两个确保”100%的成果，进一步做好“两个确保”与“三条保障线”的衔接和相关政策的配套工作。对协议期满暂时无法解除劳动关系的下岗职工，继续运用现有各类筹资渠道保障其基本生活；对已经解除劳动关系的下岗职工，按规定办理各种社会保险和接续工作。今年“低保”范围还要扩大，“低保”对象还要增加，为切实做到应保尽保，不错保、不漏保，除积极争取国家和省增加资金支持外，各级财政要进一步加大对“低保”的投入，并根据实际情况合理确定“低保”标准和补助水平。规范“低保”的申请、审批、发放和监督工作，切实防止不正之风。继续深化养老、失业和医疗保险制度改革，加强对社会保险基金的征缴，加快健全和完善社会保障体系。大力开展社会救济和社会优抚工作，建立和完善对低收入者的救助制度，对城镇中生活困难较大的家庭，在就医、子女入学和住房等方面给予必要的优惠政策和特殊照顾；认真做好受灾地区和灾民的生产自救、农业税减免及救济等方面的工作。

（八）加快财源建设步伐，做好增收节支工作

继续实施和完善扶持经济强县（区）建设、促进财政收入上台阶等加强财源建设的各项政策措施。巩固发展现有财源，高度重视培植和发展后续财源，逐步提高市级财政的宏观调控能力。继续贯彻“加强征管、堵塞漏洞、清缴欠税、惩治腐败”的方针，严格依法行政、依法治税和依法征管，切实加大稽查力度，严厉打击“偷、逃、骗、抗”税等行为，坚决制止擅自减税、免税、欠税和收“过头税”，不断完善对重点税源的征管措施，加大对零散税收的征管力度，确保财政收入稳定增长。

调整支出结构，保证重点支出需要。加强预算管理，严格执行支出预算，对财政支出做到统筹安排，确保重大决策的落实。坚决控制和压缩会议费、招待费和出国考察培训经费等一般性支出，严禁公费旅游。在确保基本工资发放、机构正常运转和社会保障支出以及各种法定支出需要的基础上，加大对农业产业化、科技教育、技术改造、环保等方面的投入力度。

深化财政管理体制改革。加快部门预算改革步伐，扩大部门预算实施范围。积极稳妥地推行国库集中收付制度改革。推进和完善乡镇零户统管工作。调整和完善县乡财政体制，重点是乡镇财政体制。完善政府采购制度，扩大采购范围，规范采购行为，提高采购资金节约率。加强对财政性资金使用情况的监督检查。加强专项资金和预算外资金管理。

（九）继续实施科教兴安和可持续发展战略，推进各项社会事业全面发展

依靠科技进步和技术创新，加快安顺星火技术密集区、黎阳高新工业园区和镇宁“星火西进示范县”的建设，加快中药材规范化种植基地建设。围绕我市经济社会发展的重点，加快科技成果向现实生产力转化，提高我市产业技术和经济竞争力。加强知识产权保护。普及科学知识，弘扬科学精神。

大力发展以“两基”攻坚为重点的教育事业。加大力度支持西秀区“两基”工作，确保通过省评估验收。继续实施“中小学危房改造工程”和“义教工程”，进一步改善农村中小学办学条件，提高办学水平。发展民族教育，整合扩大高中教育，合理布局城区中小学校，实施好现代远程教育试点工作。进一步加快高等教育发展步伐，重视职业教育，发展成人教育，努力创建安顺师范学院，办好职业技术学院、行政学院和广播电视大学。深化教育体制改革，鼓励支持和规范社会力量办学、合作办学。提高全民教育意识，加强师资队伍建设。

高度重视人才队伍建设，加大人才资源开发力度，实施人才强市战略。抓住培养、吸引和使用三个环节，建立和完善人才政策体系和工作机制，形成充满活力的用人机制。采取联合办学、委托办学等多种方式，培养和造就一大批能为经济和社会发展服务的各类人才，为全面建设小康安顺提供人才保证。

认真做好人口与计划生育工作，严格目标管理责任制和综合治理责任制，继续加强农村基层计生网络建设，加强对流动人口的计划生育管理，降低人口出生率。切实做好对资源的依法保护和合理开发利用。搞好环境污染治理。促进人口、资源、环境协调发展。

做好文化、广播电视、卫生、体育工作。推进文化体制改革，推出一批文化精品，丰富群众文化生活；加强文化市场管理；加强文化基础设施、基本队伍、基本活动内容和基本活动方式的建设。搞好农村广播电视基础设施建设，扩大广播电视覆盖面。深化医疗卫生体制和药品流通体制改革，认真做好农村和城市社区公共卫生工作，加强对重大疾病的预防控制，积极开展卫生城市（镇）创建活动；进一步健全和完善农村卫生服务网络，积极开展以大病统筹为主的新型农村合作医疗制度试点工作。提高竞技体育水平，广泛开展全民健身活动。

抓好国防教育，支持军队、武警部队和国防后备力量建设，加强军政军民团结，搞好“双拥”工作，做好人防工作。全面贯彻党的民族政策，促进民族团结和民族地区经济社会繁荣。认真执行党的宗教政策，依法管理宗教事务。认真实施妇女、儿童发展纲要，高度重视老龄工作，保护残疾人合法权益。继续做好外事侨务工作。

（十）加强精神文明和民主法制建设，切实维护社会稳定

加强社会主义精神文明建设。全面落实《公民道德建设实施纲要》，大力开展爱国主义、集体主义和社会主义教育，培育和弘扬民族精神。推进公民道德教育和道德实践，提高公民思想道德和科学文化素质，进一步形成“爱国守法、明礼诚信、团结友善、勤俭自强、敬业奉献”的良好社会风气。完善精神文明基础设施建设。开展科教、文体、法律、卫生“四进社区”活动，推进管理有序、文明祥和的新型社区建设。继续抓好文化、科技、卫生三下乡等活动。继续实施“安顺形象工程”，深入开展文明城镇、文明乡村、文明单位等群众性的精神文明创建活动，塑造安顺形象，提高市民素质。崇尚科学，反对迷信，移风易俗，加大殡葬改革力度。

加强社会主义民主法制建设，全面贯彻依法治国与以德治国相结合的方略。自觉接受人大的法律监督、工作监督和政协的民主监督。充分重视和发挥民主党派、工商联和无党派人士的参政议政作用。各项重大决策，要经过深入调查研究，充分论证，广泛听取各方面意见，由集体讨论决定。要完善政府重大决策规则和程序，努力实现决策的民主化和科学化。支持工会、共青团、妇联、残联等群众团体依法按章开展工作。加强基层民主法制建设，强化社区管理。进一步完善政务、厂务、村务公开工作。

加强社会治安综合治理，切实维护社会稳定。妥善处置突发事件和群体性事件，认真处理人民群众来信来访。巩固扩大严打整治斗争成果，加大扫除“黄赌毒”等社会丑恶现象的工作力度，增强人民群众的安全感。加强隐蔽战线斗争，维护国家安全。继续开展同“法轮功”等邪教组织的斗争。坚决落实公安部“五条禁令”，依法治警，从严治警，加强队伍建设，提高干警业务素质和执法水平，确保公正执法。加强青少年普法教育，预防青少年犯罪。认真落实安全生产责任制，以煤炭、交通、消防和农村用电为重点，加强安全生产监督和管理，切实防止重特大安全事故发生。

（十一）恪尽职守，奋发进取，切实加强政府自身建设

做好今年政府各项工作，必须以邓小平理论和“三个代表”重要思想为指导，进一步转变政府职能，恪尽职守，奋发进取，建设“廉洁、勤政、务实、高效”的人民政府。

要廉洁从政，从严治政。各级政府领导班子成员，特别是主要领导要警钟长鸣，带头廉政，自觉接受群众监督、社会监督和舆论监督。充分认识反腐败斗争的紧迫性和长期性，旗帜鲜明、毫不动摇地把反腐败斗争深入下去。认真落实党风廉政建设责任制，进一步抓好领导干部廉洁自律、查处大案要案、纠正部门和行业不正之风三项工作。坚持标本兼治、综合治理的方针，加大治本力度，加强教育，发扬民主，健全法制，强化监督，创新体制，从源头上预防和遏制腐败。严格执行“收支两条线”、招投标和政府采购制度，全面推进经营性土地使用权出让招标拍卖，积极推进产权交易进入市场。

要强化管理，依法行政。依法行政是政府正确行使行政权力的基本准则。市人民政府要在全面推进依法行政方面切实负起责任，带头依法办事。同时，要进一步加强对依法行政的领导，督促和支持各级各部门严格依法行政。要以规范执法行为、理顺行政执法体制、建立健全行政执法责任制、加强执法队伍建设为重点，全面落实依法行政的各项措施，切实增强政府系统干部职工的法制观念和依法行政能力，加强行政执法监督。积极稳妥地推进相对集中行政处罚权工作，解决多层执法和多头执法的问题。切实提高政府管理水平，努力形成行为规范、运转协调、公正透明、廉洁高效的行政管理体制。改进方式，提高效率，建立快速的信息报告反馈制度，对各项重要工作特别是重点项目建设进行“全流程”管理。进一步完善协调会议和联席会议制度，充分发挥职能部门的作用。

要转变作风，从政务实。各级政府工作人员，务必保持谦虚谨慎、不骄不躁的作风，务必保持艰苦奋斗的作风。要切实做到加强学习，与时俱进；牢记宗旨，执政为民；恪尽职守，敢于负责；求真务实，注重实效；廉洁自律，艰苦奋斗；顾全大局，团结协作。深入基层、深入群众，倾听群众呼声，关心群众疾苦，及时解决群众反映强烈的问题，真正做到重实际、说实话、办实事、求实效，形成良好的政风。建设一支既勤政务实，又廉洁奉公；既勤勤恳恳履行职责，又善于创造性开展工作；既熟悉行政管理业务，又掌握现代管理技术的公务员队伍。

各位代表！全面建设小康社会的蓝图已绘就，建设我市小康社会的目标已明确，前程似锦，催人奋进。让我们高举邓小平理论伟大旗帜，认真贯彻“三个代表”重要思想，全面落实党的十六大和省第九次党代会精神，按照市委的统一部署，凝聚全市各族人民的智慧和力量，解放思想，实事求是，与时俱进，开拓创新，为加快我市经济社会发展，实现全面建设小康社会的目标而努力奋斗！
